# Supplementary material for: Impending anthropogenic threats and protected area prioritization for jaguars in the Brazilian Amazon
Source: Commun Biol. 2023 Feb 15;6:132. doi: 10.1038/s42003-023-04490-1 (PMC9932174; doi:10.1038/s42003-023-04490-1)
Supplement: Supplementary file 2 — Supplementary Information [file 42003_2023_4490_MOESM2_ESM.pdf]

Supplementary Figures Legends

Supplementary Figure 1. Spatial distribution of 447 protected areas used to evaluate the threat to Jaguar populations across the Brazilian Amazon.

Supplementary Figure 2. Linear relationship between jaguar population size inside PAs (derived from Jędrzejewski et al. 2018[25] density estimates) and conservative jaguar population size inside PAs (derived from density average – 1.*se*, and them categorizing in density classes (i.e. 0.00 = <0.01; 0.01 = 0.01-0.02; 0.02 = 0.02-0.03; and 0.03 = >0.03)).

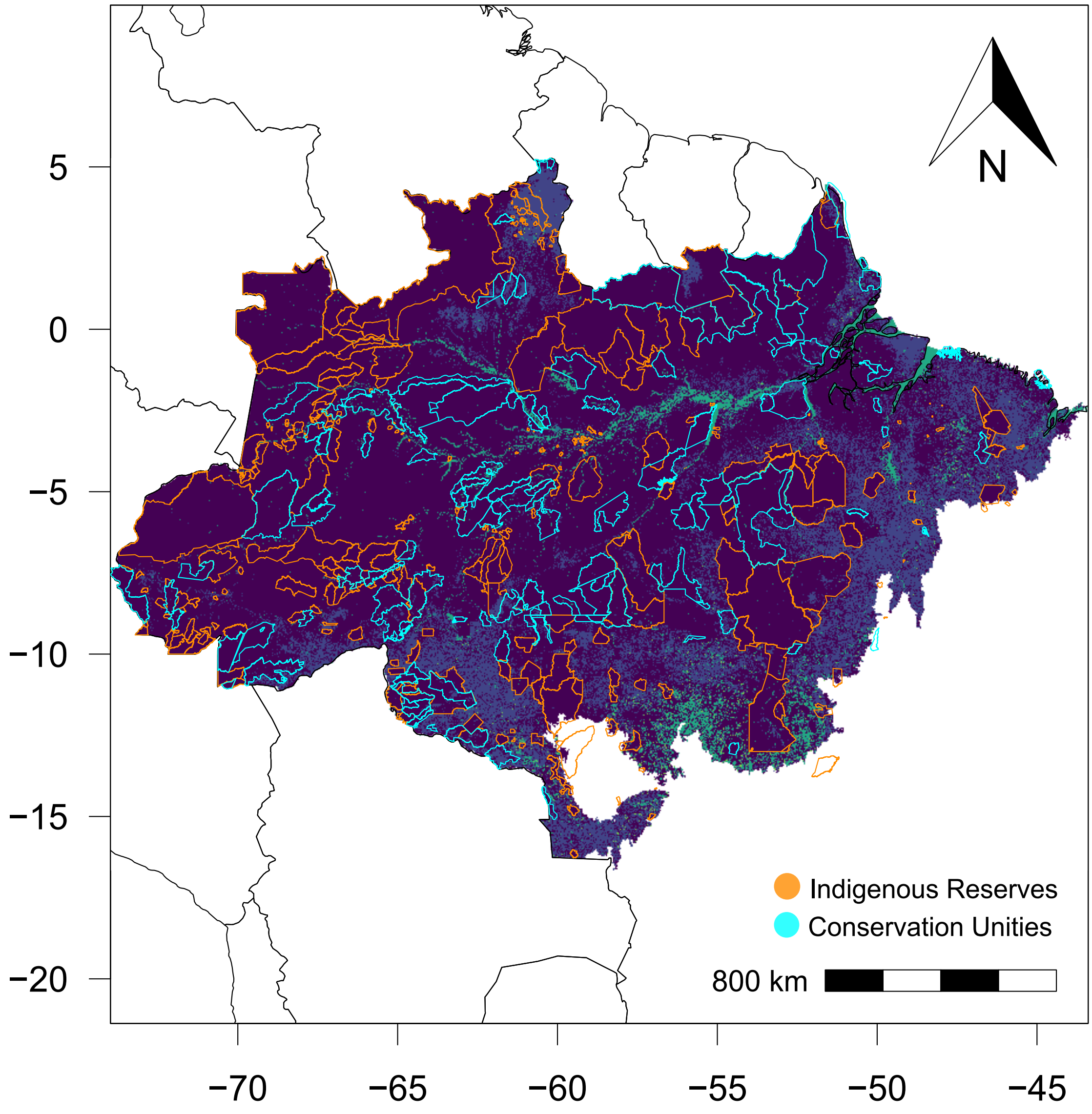

5
0
−5
−10
−15
−20
−70
−65
−60
−55
−50
−45
N
Indigenous Reserves
Conservation Unities
800 km

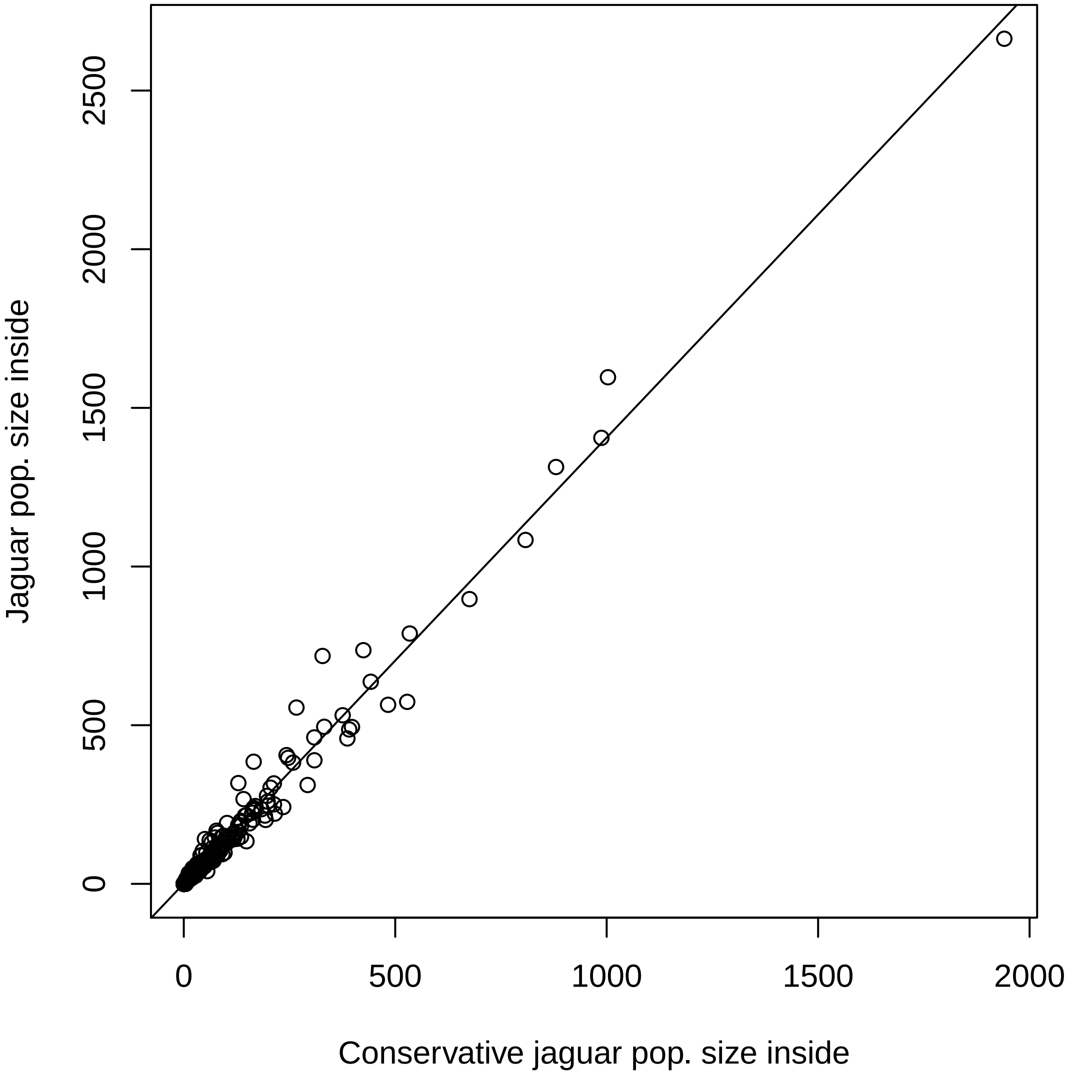
Jaguar pop. size inside
Conservative jaguar pop. size inside
0
500
1000
1500
2000
2500
0
500
1000
1500
2000
