# Supplementary material for: Impending anthropogenic threats and protected area prioritization for jaguars in the Brazilian Amazon
Source: Commun Biol. 2023 Feb 15;6:132. doi: 10.1038/s42003-023-04490-1 (PMC9932174; doi:10.1038/s42003-023-04490-1)
Supplement: Supplementary file 3 — Description of Additional Supplementary Files [file 42003_2023_4490_MOESM3_ESM.pdf]

Description of Additional Supplementary Files

File name: Supplementary Data 1

Description: Sociopolitical details of 447 protected areas used to evaluate the threat to Jaguar populations across the Brazilian Amazon. Acronyms are: SPA: strictly-protected conservation units; SUR: sustainable use conservation units (SUR); IR1: declared Indigenous Reserves; and IR2: Indigenous Reserves that were delimited, approved, or ratified.

File name: Supplementary Data 2

Description: ANOVA and post-hoc Tukey results comparing socio-environmental variables between protected area types across the Brazilian Amazon. SPA: strictly-protected conservation units; SUR: sustainable use conservation units (SUR); IR1: declared Indigenous Reserves; and IR2: Indigenous Reserves that were delimited, approved, or ratified.

File name: Supplementary Data 3

Description: List of 74 additional protected area codes, names, size (km2), and legal status of high-priority reserves for jaguar conservation across the Brazilian Amazon. Acronyms are SPA: Strictly Protected Conservation Units; IR2: Indigenous Reserves that were delimited, approved, or ratified; and TI: threat index. See the prioritization approach in Fig. 3C.

File name: Supplementary Data 4

Description: Complete list and dataset of socio-environmental variables for 447 protected areas across the Brazilian Amazon extracted based on geospatial layers (see Methods). Acronyms are SPA: Strictly Protected Conservation Units; IR2: Indigenous Reserves that were delimited, approved, or ratified; and TI: threat index.

File name: Supplementary Data 5

Description: Summary of socio-environmental variables (contained in the Supplementary Data 4) for 447 protected areas across the Brazilian Amazon extracted based on geospatial layers (see Methods). Acronyms are SPA: Strictly Protected Conservation Units; IR2: Indigenous Reserves that were delimited, approved, or ratified; JO: jaguar outside PAs; JI: jaguar inside PAs; and TI: threat index.
