# Supplementary material for: Impending anthropogenic threats and protected area prioritization for jaguars in the Brazilian Amazon
Source: Commun Biol. 2023 Feb 15;6:132. doi: 10.1038/s42003-023-04490-1 (PMC9932174; doi:10.1038/s42003-023-04490-1)
Supplement: Supplementary file 9 — Reporting Summary [file 42003_2023_4490_MOESM9_ESM.pdf]

nature research

Corresponding author(s): JULIANO A. BOGONI - COMMSBIO-22-0925B

Last updated by author(s): Dec 17, 2022

Reporting Summary

Nature Research wishes to improve the reproducibility of the work that we publish. This form provides structure for consistency and transparency in reporting. For further information on Nature Research policies, see our Editorial Policies and the Editorial Policy Checklist.

Statistics

For all statistical analyses, confirm that the following items are present in the figure legend, table legend, main text, or Methods section.

n/a	Confirmed	
☐	☒	The exact sample size (*n*) for each experimental group/condition, given as a discrete number and unit of measurement
☐	☒	A statement on whether measurements were taken from distinct samples or whether the same sample was measured repeatedly
☐	☒	The statistical test(s) used AND whether they are one- or two-sided<br>*Only common tests should be described solely by name; describe more complex techniques in the Methods section.*
☐	☒	A description of all covariates tested
☐	☒	A description of any assumptions or corrections, such as tests of normality and adjustment for multiple comparisons
☐	☒	A full description of the statistical parameters including central tendency (e.g. means) or other basic estimates (e.g. regression coefficient) AND variation (e.g. standard deviation) or associated estimates of uncertainty (e.g. confidence intervals)
☐	☒	For null hypothesis testing, the test statistic (e.g. *F*, *t*, *r*) with confidence intervals, effect sizes, degrees of freedom and *P* value noted<br>*Give P values as exact values whenever suitable.*
☒	☐	For Bayesian analysis, information on the choice of priors and Markov chain Monte Carlo settings
☒	☐	For hierarchical and complex designs, identification of the appropriate level for tests and full reporting of outcomes
☒	☐	Estimates of effect sizes (e.g. Cohen's *d*, Pearson's *r*), indicating how they were calculated

Our web collection on statistics for biologists contains articles on many of the points above.

Software and code

Policy information about availability of computer code

Data collection

We used several high-resolution spatial layers (rasters or polygons) to extract variables for each protected area that represent (1) a proxy of direct jaguar mortality (e.g. roadkills and persecution due to livestock depletion52): (i) human population density (HPD), sourced from the Brazilian Institute of Geography and Statistics (spatial scale of 1:250,000)77; (ii) road density (including paved and unpaved roads), sourced from the Brazilian Institute of Geography and Statistics (spatial scale of 1:250,000)78; (iii) pasture area (also considered as habitat degradation), sourced from MapBiomas (v.5, spatial resolution of 30 m)79; and layers that represent (2) habitat loss and degradation: (i) fire hotspots over a 5-yr period (2016 - 2020), sourced from the National Institute for Space Research-INPE (TERRA satellite MODIS sensor; 1-km spatial resolution)34; (ii) deforestation over 4 years (2016 - 2019) sourced from PRODES (30-m spatial resolution)34; and (iii) mining areas, sourced from MapBiomas (v.5, 30-m spatial resolution)79. We also obtained the size of each protected area and its adjacent 5-km buffer zone. Based on the SIRGAS-2000 UTM-ZONE 22°S projection, spatial data extraction was performed separately for both the internal PA area and the external 5-km buffer based on the administrative polygons of each protected area. We used a conservative 5-km buffer threshold because this is approximately the minimum radius for the home range of Amazonian jaguars (i.e. 4.7 km for females, ca. 79 km2 considering a radial-buffer)12,22. Given that the average is a radius of 6.7 km12,22, the conservative 5-km buffer represents an additional area of 448,452.72 km2 (8.7% of Brazilian Amazon). We sourced data on jaguar population density inside each protected area from Jędrzejewski et al. (2018)27. Data extraction was conducted using the ArcGIS 10.8 software80 based on the average or sum of pixels/area both inside and outside each PA, independently of spatial overlap (pixel vs. PAs) area. Further, we obtained the type of legal denomination of each protected area (according to Sistema Nacional de Unidades de Conservação (SNUC64), based on Ministério do Meio Ambiente (MMA81), and the stage of legal implementation of each indigenous reserve (i.e. declared, approved, physically demarcated, and legally sanctioned) sourced from Fundação Nacional do Índio82.

Data analysis

Jaguar density bias and buffer size evaluation
We formally evaluated the predicted jaguar population densities across all Amazonian PAs as derived from Jędrzejewski et al. (2018)27 through: (1) a descriptive exploration of the standard errors (se) derived from each pixel inside PAs and their respective 5-km buffer; (2) contrasting the predicted values of jaguar density from Jędrzejewski et al. (2018)27 with in situ estimates based on field studies across the

Neotropics previously compiled by Tobler and Powell83; and (3) comparing jaguar densities at 13 sites within and immediately around Brazilian Amazonia based on both published (see de Oliveira et al. (2012)84, Ramalho (2012)85, Duarte et al. (2022)86) and unpublished data (Morato et al. unpublished data) and values within a 5-km radial buffer at the same geographic coordinates derived from Jędrzejewski et al. (2018)27. To further assess buffer sizes, we also extracted the jaguar density estimate within a 10-km buffer, which were then regressed against those within a 5-km buffer.

Jaguar population responses to threats and threat index (TI) for protected area prioritization criteria
We tested for differences among PA types (i.e., IR1, IR2, SPA, SUR) in how the main response variable (jaguar population size) responded to our environmental predictors (see below) using ANOVAs followed by Tukey post-hoc comparisons by correcting for data asymmetry using log10 (x + 1)87. We constructed a "threat index" (TI) applied to each of the 447 protected areas using the above geospatial layers for both each PA (in) and each respective buffer polygon (out), which are weighted according to specialized literature on jaguar threats (see 4,16,25,27,48, 88-92). For instance, the major causes of jaguar declines is a synergistic effect of habitat loss, fragmentation, and killings (generally linked to human population density) (e.g. Paviolo et al. 200825, Medellín et al. 200288, Quigley et al. 201589, Bogoni et al. 202290, Valsecchi et al. 202291), therefore, these variables received the largest weight in our TI, whose sum can be larger than 1.0 due to synergetic effect upon mammal populations90. Yet, other major causes such as roadkill, mining and wildfires frequency and severity also impact directly jaguars across the tropics92 but comparatively low — until now — than deforestation25 and killing91.
To do so, the TI incorporated the following variables calculated for both the PAs ("inside") and their 5km buffer areas ("outside"): (1) ratio of mining threats (min), defined as the size of mining operations (km2) in relation to PA size (km2), (2) pasture area (pas) defined as the size (km2) of pasture areas both inside and outside PAs, (3) ratio of deforestation area over a 4-yr time-series (def), based on the amount of cumulative deforestation (km2) in relation to PA size; (4) total length (km) of roads (roa) overlapping each PA; (5) density of fires (fir) defined as the fire frequency over the 5-yr time-series divided by the PA size; and (6) the maximum human population density (hpd) for each polygon area. We thus assigned relative weights to these variables to compose the TI according to literature, weighting the threats inside PAs asymmetrically in comparison with the threats outside (i.e. 0.65 vs. 0.35), given that PAs have irreplaceable roles to retain the biodiversity93. We also rescaled the threat index given the maximum value at any protected area, which therefore ranged from 0 to 1 by dividing any TIi for the max TIi,j. The threat index — ranging from 0.0 to 1.0 — was obtained given the following equation:

〖TI〗 _(i-protected area)=(■(0.65 ×(∑▒(〖min〗 _in× 0.05+ 〖pas〗 _in× 0.25+ 〖def〗 _in× 0.50 + 〖roa〗 _in× 0.10+ 〖fir〗 _in × 0.15+ 〖hpd〗 _in×0.35))@+ 0.35 × (∑▒(〖min〗 _out× 0.05+ 〖pas〗 _out× 0.25+ 〖def〗 _out× 0.50 + 〖roa〗 _out× 0.10+ 〖fir〗 _out × 0.15+ 〖hpd〗 _out×0.35)))/ 〖maxTI 〗 _(i,j))

To identify PAs with the highest priority for short-term jaguar conservation action, we constructed a bivariate plot between jaguar population sizes inside any PA vs. the threat index (TI). To obtain the jaguar population sizes inside PAs, we used the more conservative value of jaguar density estimates sourced from Jędrzejewski et al. (2018)27 (i.e. density average – 1.se) and then categorizing them into population density classes (i.e. 0.00 = <0.01; 0.01 = 0.01-0.02; 0.02 = 0.02-0.03; and 0.03 = >0.03) to reduce the uncertainty of the estimates. Based on the average of both variables at the bivariate plot, we defined one quadrant of short-time high priority (ST-HP) based on large conservative jaguar populations confronting high threat indices. We then identified the top-10 PAs for which conservation efforts should be allocated across the Brazilian Amazon, selecting areas located in the extreme distribution across the ST-HP quadrant by adding a tangential line within the quadrant that separates the top-10 areas in terms of largest jaguar population sizes vs. highest TIs. Once we identified the main spatial covariates related to jaguar population sizes, we then tested differences between high- and low-priority PAs using an ANOVA followed by Tukey post-hoc tests while correcting for data asymmetry using log10 (x + 1)87.

For manuscripts utilizing custom algorithms or software that are central to the research but not yet described in published literature, software must be made available to editors and reviewers. We strongly encourage code deposition in a community repository (e.g. GitHub). See the Nature Research guidelines for submitting code & software for further information.

Data

Policy information about availability of data

All manuscripts must include a data availability statement. This statement should provide the following information, where applicable:

- Accession codes, unique identifiers, or web links for publicly available datasets
- A list of figures that have associated raw data
- A description of any restrictions on data availability

Data and R-code are available as supplementary file.

Field-specific reporting

Please select the one below that is the best fit for your research. If you are not sure, read the appropriate sections before making your selection.

☐ Life sciences ☐ Behavioural & social sciences ☒ Ecological, evolutionary & environmental sciences

For a reference copy of the document with all sections, see nature.com/documents/nr-reporting-summary-flat.pdf

Ecological, evolutionary & environmental sciences study design

All studies must disclose on these points even when the disclosure is negative.

Study description	Based on geospatial layers, we extracted socio-environmental variables for 447 protected areas across the Brazilian Amazon to identify protected areas that merit short-term high-priority efforts to maximize jaguar persistence.
Research sample	We used several high-resolution spatial layers (rasters or polygons) to extract variables for each protected area that represent a

Research sample	proxy of direct jaguar mortality and layers that represent habitat degradation (see Methods).
Sampling strategy	We scoped this study to include all officially sanctioned protected areas across the Brazilian Amazon, including 117 conservation units and 330 indigenous reserves, amounting to 1,755,637 km2.
Data collection	Data extraction was conducted using the ArcGIS 10.8 software based on the average or sum of pixels/area both inside and outside each PA.
Timing and spatial scale	Current; Brazilian Amazon
Data exclusions	No
Reproducibility	The data extraction and analysis are reproductible for all regions that have high-resolution spatial data.
Randomization	This study include all officially sanctioned protected areas across the Brazilian Amazon, therefore, no randomization was necessary.
Blinding	This study include all officially sanctioned protected areas across the Brazilian Amazon, therefore, no blinding was necessary.

Did the study involve field work? ☐ Yes ☒ No

Reporting for specific materials, systems and methods

We require information from authors about some types of materials, experimental systems and methods used in many studies. Here, indicate whether each material, system or method listed is relevant to your study. If you are not sure if a list item applies to your research, read the appropriate section before selecting a response.

Materials & experimental systems

n/a	Involved in the study
☒	☐ Antibodies
☒	☐ Eukaryotic cell lines
☒	☐ Palaeontology and archaeology
☒	☐ Animals and other organisms
☒	☐ Human research participants
☒	☐ Clinical data
☒	☐ Dual use research of concern

Methods

n/a	Involved in the study
☒	☐ ChIP-seq
☒	☐ Flow cytometry
☒	☐ MRI-based neuroimaging
